# When Pay-Day Comes

**Published:** 1897-07

**Authors:** 


### When Pay-Day Comes.

There is a large number of persons who do not hesitate to run up a large amount at the stores, provided they can have things charged. They forget that one of these days there must come a settlement.

There are certain drugs which have such an effect on one's nervous system that work appears lighter, therefore he is induced to exert himself still further and draws upon his reserve force to a marked degree. To be sure, the nervous system is so affected that he is not aware of this tax on his reserve powers.

It is a serious thing for us to take any remedy which will keep us from being warned that we need rest. Some months since, we had occasion to remark in these columns that there was hardly an athlete or bicycle rider who laid any claims to endurance who did not carry a good supply of those drugs which were said to increase the amount of muscle power. The day of reckoning is sure to come, and unless the excessive use of these drugs be checked we will have our strongest and best young men develop into dyspeptic, nervous and irritable wrecks.—*Pract. Med.*

If the devil punishes the ordinary harmless liar, what will he do with the dentist who advertises to extract teeth without pain?
—*Med. Era.*

